# Observations on the Principal Hospitals for the Insane in Great Britain, France, and Germany

**Published:** 1846-10-01

**Authors:** 


### Observations on the Principal Hospitals for the Insane in Great Britain, France, and Germany. By *J. Ray*, M.D. [American Journal of Insanity, 1846.]

The criticisms and opinions of enlightened foreigners who have viewed our institutions with an impartial and appreciating eye, form the most valuable and welcome returns for any attentions they may have received while among us. It is on this account we purpose laying the substance of Dr. Ray's essay before our readers, as a production written with great good feeling, and by one who, from his office of superintendent of an asylum in his own country, is fully qualified to offer an authoritative opinion upon what he has observed in this. Perhaps we are scarcely justified in styling our Transatlantic brethren foreigners; for, however great the political hostility and animosity which have at times prevailed among the mass of the inhabitants of either country, the medical profession has always constituted itself a neutral ground upon which all may meet with that brotherly feeling fitting to be exhibited by the descendants of one common parent. With one exception, indeed, the reciprocal professional relations are as satisfactory as can be desired, and we need hardly state that the non-existence of an international copyright constitutes that exception. Its absence is far more injurious to the Americans than to ourselves. It is true that we are the pecuniary losers by it, and it can hardly be expected that a writer connected with a Journal, which, like ours, has been systematically pirated for years, can look at this element of the question with indifference ; but the truly important aspect in which it should be regarded is the depressing effect which it exerts upon American medical literature. While numberless English works are reprinted on the other side of the Atlantic as soon as they appear, some two or three sterling productions are all our publishers, with equal facilities, have thought it worth their while to re-issue. And how can it be otherwise than that a nation which systematically relies for the supply of its literary food upon what it begs or filches from others, must be destitute of the stimulus and vigour necessary for producing it itself. Take away English re-prints and translations from the French, and verily a small book-case will contain the productions of American medical (as indeed general) literature. Of the capabilities of American medical practitioners, none who have perused the original articles in their Journals, or the few good works they have published, can entertain any doubt. We there find the same practical sagacity which we are proud to say characterizes the profession in our own country : but the results can never be exhibited in the form of good books, commanding an European reputation, until the vicious system of living upon the fruits of other person's labours has been discontinued. It is bad enough that no international copyright should exist between nations speaking different languages, but that it should be wanting amid people speaking the same tongue and boasting a common origin is indeed disgraceful.

To return to Dr. Ray's Essay. He availed himself of one of those opportunities which so rarely fall to the lot of medical men—a few months leisure—for the investigation of the condition of the principal insane establishments in England and France and some of those in Germany. For the information of his countrymen he has published the results of his tour in the very interesting and useful "Journal of Insanity," conducted by Dr. Brigham, and institutes a comparison between the institutions he had just visited, and those of his own country, assigning the following reason for so doing :—

"Presuming that the results of my observations may not be entirely devoid of interest to my professional brethren, and perhaps to some others, I am induced to offer them to the public, hoping that they may be productive of good in a department of philanthropy where much yet remains to be learned. If we would have our course in it characterized by progress, it will be better to dismiss all notions of superior excellence, learn what others are doing, and be willing to receive with a teachable disposition the lessons they offer us. I fear we have been too prone to believe that our institutions for the insane are far beyond those of any other country, and in a spirit of self-complacency, have gone on year after year copying one another—too often our faults and merits alike—scarcely evincing a suspicion that anything could be learned from abroad. We do not seem to have been aware that in Europe these institutions, for the last few years, have received a large share of public attention, and that intellectual effort and enlightened philanthropy have been devoted to their improvement, to a degree quite unparalleled in America. We should recollect that, there the greater control of the Government over all matters pertaining to the public good, and the greater wealth of the community of England, if no where else, give them an immense advantage over us in improving the condition of the public charities. Here the let-alone-policy, which we have rejected in regard to trade, has been too much adopted in measures of philanthropy, which are thus abandoned to individuals whose zeal may not be according to knowledge, and whose pecuniary means are inadequate to carry out their undertakings in a generous and lofty style. Their steady, systematic, and intelligent mode of proceeding strongly contrasts with the fitfulness, irregularity, and lack of intelligence that characterize so many of our benevolent efforts, and produce an imperfect and disjointed result. It would not be strange then if it should appear that, in many respects, we have been outdone in a field of benevolent and scientific exertion where we have flattered ourselves that we shine without a rival."

We shall notice the various heads under which Dr. Ray has classified the results of his observations.

1. *The Visitation and Direction of Asylums.*—Under this head we find some well-deserved censure upon the system which prevails among us. Who that has had an opportunity of witnessing the meddlesome inefficiency of visiting Justices of the Peace, and Governors of Subscription Asylums and Hospitals but must acknowledge the truth of this passage.

"The small share of the individual in the general interest, is swallowed up in the more direct and personal interest that springs from his official relations. Most of them can have but a vague idea of the duties of their office, yet they are naturally pleased with the power which it confers, and especially that kind of it most pleasing to a certain class of minds—the power of patronage. Delegating no power which they can possibly exercise themselves, and constantly hampering those whom they have entrusted with any, it has come to pass that the English asylums have probably been directed with less intelligence and disregard of unworthy considerations than any on the Continent, or in America. The visiting Justices have been described by one who has seen much of them as 'on the subject of insanity profoundly ignorant, individually irresponsible, and collectively despotic.' Indications of the spirit in which they exercise their functions, may be gathered from the rules they have made; and it is a spirit which can form no higher opinion of the duties and characters of those who are charged with the care of the insane than by regarding them like those of the keepers of jails, workhouses and prisons. * * * * * * * * * * In some establishments the visiting justices even participate in the executive management, upon the idea that none can be trusted but themselves. At Hanwell, they furnish themselves with keys and enter the wards, changing attendants and patients from one gallery to another, and dispensing their orders with the utmost freedom. It is not strange, that under such management, this institution should have been filled with confusion and disorder: nor can we wonder at the remedy such persons adopted, to counteract the effect of their own mischievous interference. Conceiving that a little military discipline would meet the exigency of the case, they installed into the office of Superintendent, a half-pay military officer. Of course, this arrangement could not last long, but I could not learn that any wisdom was learned from such experience."

When we consider the conduct of these meddling bodies we cannot but admire the tact with which Dr. Conolly managed to carry on his great experiment at Hanwell, and still more to wonder at the success which attended it. And yet, after all, he was obliged to retire from the management of an establishment in which it would be difficult to say whether the prevalence of vexatious opposition or injudicious co-operation has proved most detrimental to true progress. It becomes indeed a serious question whether the well-being of the insane is much longer to depend upon the remote contingency of men of common sense being elected on the Visiting Boards, who, after assuring themselves of the capacity of their Medical Superintendent, will be content to leave the absolute management of the patients in his hands. That we are far from this point many parts of Dr. Ray's Report too clearly exhibits. Even so apparently an essential power as the dismissal of servants improperly conducting themselves is not placed in the hands of the medical superintendent, and in more than one instance they have been retained, although protested against as unfitted for their situations! Dr. Ray, too, well observes, that the whole tenour of the regulations of most asylums implies a necessity of a scrutinizing surveillance of the conduct of the superior officers of these establishments exceedingly derogatory to them. Hence, a servant not liable to dismissal by the superintendent is supposed to be more at liberty to report any misconduct of the latter that may come under his cognizance. "The suspicion is never to be dismissed that these gentlemen are ready to take advantage of any opportunity to go wrong, and need to be hedged in by a system of checks and balances. The Superintendent is very much regarded in the light of an upper-servant of some cleverness and honesty, rather than of a gentleman whose talents, moral worth, and scientific attainments have raised him to a highly responsible, arduous, and honorable position in his profession."

A rule is quoted from among those adopted at the Lincoln Asylum, and well characterized as mischievous in its tendency, as indicative of inconceivable meanness in those who made it. "Each patient discharged recovered shall be questioned by a deputation of the Board, not only with respect to the treatment of himself or herself, but also as to the treatment of the other patients." Those who know more of the medical institutions of the city of Lincoln than a casual visitor can, will feel surprised, not at this or any other absurdity being perpetrated, but that any improvement in the management of the insane should ever have been there first set on foot.

In France, the medical officers are selected from a body whose preliminary training has thoroughly qualified them for their occupation. They are appointed by the Minister of the Interior from a list of three nominated by the General Council; and the chief medical officer of the establishment exercises a paramount authority in everything regarding the interests of the patients.

2. *Officers*.—We quite agree with Dr. Ray that the distribution of duties is far more rational and efficient in America and Germany than in France and England. In the first-named country the Superintendent or *Resident Physician* is invested with paramount authority in everything which relates to the management of the patients, as also with the power of appointing and discharging the attendants. An assistant physician shares his labours and seconds his views. The duties of the Matron are limited to the management of house-keeping, and the superintendence of the work and clothing of the female patients. How different from this simple common-sense plan is our own.

" There the officers are entrusted with less power, and that is more equally divided between them. Thus, responsibility is frittered away, and that unity of plan and purpose so necessary in maintaining the ordinary routine of service, not to speak of any higher end, is entirely wanting. In one of the largest establishments of the country I found that the officers themselves were not quite clear as to the exact limits of each other's responsibility. If the resident physician prescribed medicine, his prescription was liable to be countermanded by the visiting physician: if he moved a patient or attendant from one gallery to another, the matron might move them back again; and if the matron shut up a patient in her room, the visiting justices might, and probably would, order her to be enlarged. Thus each one was constantly interfering with somebody else, and preparing for some fresh jealousy or heart-burning, disorder or dissatisfaction. * * * * * * * * *
It was not perceived that there should be a radical difference between the management required for patients labouring under diseases of the body, and that most suitable for persons smitten with disorders of the mind.

To this cause is chiefly to be attributed the slow progress which institutions for the insane have made in the way of improvement. The officer in the immediate charge of the patients had the least power to minister to their moral necessities, even if capable of perceiving and appreciating them, while the one who had the power could see nothing in his hasty and infrequent visits to call for its exercise. Even after the gross abuses that resulted from this management were unfolded, it was attempted to remedy them, not by cutting off the source of the evil, by putting the establishment into the charge of men possessing moral and scientific qualifications of a high order, and invested with the necessary power for carrying their views into effect, but by making stricter rules and a more searching espionage."

In nearly all the English establishments there is indeed a resident officer of some kind, but there is also one a mere visiting physician, and the respective duties of these officers are very loosely defined and very different in different institutions. " In some, the visiting physician seems intended for ornament rather than use, his visits being a mere matter of form, while the resident medical officer is the real chief. In others, the visiting physician is the fountain of all power the Justices have not kept to themselves, and directs the whole management, the duties of the resident medical officer being confined to the execution of his orders, which may be so general that the former reap all the credit of their success, and avoid any responsibility that may be incurred by their failure. In the rules of the Lincoln Asylum the House-Surgeon is expressly forbidden to enter the galleries of the female patients without the attendance of the matron or some responsible female!!" In a note, Dr. Ray furnishes from the pen of a friend a graphic account of the ludicrous farce performed at a *weekly* visitation of the physician of St. Luke's, during which patients were prescribed for at the rate of about fifteen per hour, the peculiarities, habits, &c., of those unfortunate individuals being enquired into in their presence, and in that of their comrades in misfortune.

The *Matron* is oftentimes a too important, and even a mischievous person, in the English asylum. " In some establishments, she is the principal member of the government on the female side of the house, completely controlling the same, and yielding to the physician only in the matter of medical treatment." No one can doubt the great utility of female influence in the management of the insane, but, for this end, it must act implicitly under the superintenda[illegible]e and control of the medical officer, and never in contravention to his commands. How seldom it is the case that a matron will consent to this, all who know much of our public asylums must be aware. Immense benefit would often accrue from appointing the wife of the medical superintendant to the office.

In France, the chief medical officer possesses paramount power, but is not resident. He passes a much longer period among the patients than the visiting physician does in England. His non-residence is however a very serious defect.

Generally speaking, the officers of European asylums are not so well remunerated as are those of the establishments in America.

3. *Site of European Asylums.*—Dr. Ray speaks with great and well-deserved admiration of the site of many of our county-asylums, and the tastefulness with which the grounds surrounding them are laid out. By far the most beautiful establishment, however, that he had the opportunity of visiting was, that of MM. Falret and Voison at Vanves, near Paris, situated in the midst of 100 acres, laid out in the finest style of landscape gardening. The Americans, notwithstanding their unlimited command of land, are far less liberal in their devotion of it to the purposes of asylums. While gladly acknowledging the admirable situations and external arrangements of our county asylums; we must not forget the pitiable state, in this respect, of various other establishments. We allude to private and public institutions situated in London and other towns, nearly, and in some cases totally, destitute of the means of affording the patient, not a fine prospect, for the circumstances of the locality may generally render that impossible, but even more room for exercise than a wild beast possesses in a menagerie. It is notorious that this is the case with several establishments, and it surely would be no injustice to compel those proprietors who are unable to furnish the requisite accommodation in this vitally important respect either to discontinue their occupation or to remove to more convenient localities.

4. *Construction,*—Dr. Ray admires the more recently constructed asylums in Britain, both for their convenience and appearance, especially those, such as the Surrey and Glasgow, which are erected in the Tudor-Gothic style. He considers that it is far better to extend the erections for the obtaining the requisite room than to raise them above two stories in height. The interior of the asylums he visited favourably contrasted with those in America in their greater amplitude, the width of the halls and corridors, the height of the ceilings, the spaciousness of the day-rooms, and in their light and cheerful aspect. The rooms are, with few exceptions, placed only on one side of the galleries, while in America the vicious plan of constructing them on both sides prevails.

5. *Sleeping-Rooms and Dormitories.*—The pauper sleeping-rooms in the

English asylums, Dr. Ray states, have a naked, cheerless, prison-like appearance; but he adds, "It would be a misplaced kindness to furnish poor patients with accommodations very much better than they ever knew before, and with which their own poverty-stricken abode, when they return to it, would present a painful contrast. Wisely, therefore, have cleanliness and suitable comfort been regarded as the only essential requisites in these rooms."

The directors of the different European establishments entertain very different opinions upon the expediency of large *associated dormitories.* In England, these seldom contain more than a dozen beds, and often a much less number; while, in France, they sometimes have 50 placed in them. A very much larger proportion of the patients sleep in dormitories in the latter country than in England. The dormitory system has been much disapproved of by some as calculated to allow of annoyance by noisy or filthy patients; but those who have most employed them maintain that one of their chief advantages consists in the greater quietude which many excited patients assume when placed in them. This testimony was afforded the author in France and Scotland, and at Siegburg. In America, where every patient has his room which he may call his own both by day and night, the general introduction of such dormitories would be impossible. Moreover, the much larger proportion of excited patients would prevent this. Still the author is convinced, that in certain cases they would prove very useful.

"There is a class of timid, nervous patients, who would be far more comfortable in an associated dormitory, especially when they first enter the institution. A poor, nervous female, startled by every sound, and apprehending every possible evil, who finds herself, perhaps for the first time for years, shut up in a room by herself, with no companion but her own agitated thoughts, unable to sleep, afraid to speak, is thrown into a state of disquietude, sometimes amounting to agony, not very favorable to recovery. Here the difficulty would be completely met by an associated dormitory. They are far better also for those suicidal cases which we now manage by having an attendant sleep in their rooms by the side of the bed. To be obliged to sleep on the floor in a room barely large enough for one, has no tendency to improve the humour of the attendant, and the consciousness of being specially watched is not likely to exert a salutary effect upon the mind of the patient. Nothing in the management of an asylum is a source of so much embarrassment as that class of patients in whom we suspect the existence of a suicidal disposition, but which is so feeble, or so successfully concealed, that we hesitate to place an attendant in their room at night, from the fear of unnecessarily exciting displeasure, and even suggesting suicidal thoughts where they did not previously exist. Thus, the patient goes unwatched, until he is found some morning ingeniously strangled by his pockethandkerchief, or a strip of his sheet. The liability of such deplorable events is much diminished by the use of dormitories. I do not mean to say that suicide would never occur in a dormitory, for the disposition is sometimes so strong that nothing short of unremitting vigilance can prevent it. What I do mean is, that it would be effectually prevented in that large class of the suicidal in which the propensity is not so strong as to lead to its gratification in the presence of others, whether asleep or awake. Many filthy patients, too, when tranquil, are, no doubt, better managed in dormitories, because the supervision there exercised is sufficient to improve their habits by exciting their self-control, and also secures that attention to their wants which they cannot receive so effectually when sleeping alone. * * * * * * * The dormitory itself, when tastefully fitted up, presents a more cheerful and pleasing appearance than rows of small solitary rooms, which necessarily have a somewhat prison-like appearance. Few features in the foreign asylums left a more pleasing impression upon my mind than some of their dormitories. Those of the Northampton asylum were the very embodyment of good taste, neatness, and propriety—temples to the somniferous god in which it would seem he must be delighted to dwell. In walking over the Salpetrière I could not help stopping repeatedly to admire the *coup d'œil* which one obtained by standing in the door-way of one of its dormitories, and looking towards its opposite side."

6. *Day Rooms.*—Little or nothing has as yet been done, either in America or Europe, to relieve the nakedness of the walls of these by the suspension of maps, pictures, &c.; although they should be rendered as attractive as possible in order to induce the patients to resort to them, and prevent the baneful practice of remaining alone. The substitution of long, narrow, half-lighted galleries for cheerful day-rooms is a great error. The *windows* of the rooms in the British asylums too often have a very prison-like appearance. They are also superfluously guarded, and in a far greater degree than prevails in France. In Edinburgh and Glasgow a very convenient shutter is employed for the windows of excited patients, which, out of sight at other times, may be made very conveniently when required to cover any portion of the window desired. The *doors* of European asylums are also closed, and locked with far more clatter and noise than in America, owing to the ponderous nature of the instruments employed. The small tubular key described by Jacobi is in universal use in the States.

7. *Padded Rooms.*—These much excited the author's curiosity, as he felt convinced, from the description he had heard of their utility, either that insanity is a different thing in England from what it is in America, or that he had not been rightly informed of the nature of the cases in which they are employed. Subsequent enquiry confirmed these opinions. In England even, however, opinions are divided as to the utility of these chambers, but the author is disposed to believe they may be of much more service in this country than in America, where the violence in cases of excitement is so much greater.

8. *Warming and Ventilation.*—The author was much surprised not only at the want of system of ventilation in the English asylums, but at the little importance their directors seemed to attach to its absence. They, for the most part, seemed content to rely upon the simple expedient of opening the windows, an imperfect one at all times, and impracticable in cold and wet weather. The infected state of the large dormitories was generally complained of, and stated erroneously as one of their inherent defects. Dr. Ray is a great admirer of Dr. Reid's system of ventilation, but with our present experience of its inefficiency it is not very likely to be introduced into any new establishment in which the preservation of health is an object in view.

9. *Attendants.*—In America, owing to the ease with which more agreeable employment may be obtained elsewhere, attendants will not remain more than a year or two in the establishments, while in England they are too often found therein after they have lost all their efficiency. In France, an excellent classification of the attendants prevails. One class performs the menial services, another acts as companions to the insane, and executes the orders of the remaining or superintending and responsible class, the organs of communication between the patients and the officers. It is obvious that much of the efficiency of the asylum depends upon the qualifications of the immediate attendants, and we fear the remuneration offered by most of our asylums is not sufficient to ensure these being of a high order.

10. " *Quiet of the European Asylums.*—With no feature of the foreign asylums was I so forcibly struck as the extraordinary quiet of the patients, as contrasted with the uneasiness and agitation of ours. Not a single instance of vociferation did I witness, and cries, and shrieks, and shouting, I rarely heard. That peculiar kind of vituperation joined with inexhaustible volubility, so familiar to those who have had charge of the insane on this side of the Atlantic, seemed to be unknown there; and even in the refractory wards, instead of the agitation and disorderly movements that characterize that class with us, there was a degree of stillness and quiet that would lead one to suspect, at first thought, that he was among the tranquil or convalescent. A visitor in passing through one of our asylums is beseiged by persons who fill his ears with the bitterest complaints, representing themselves as the victims of the grossest injustice, and importuning him to procure their discharge. He is almost disposed to believe that there is really something wrong; that such feelings must have some other foundation than mere fancy; and he needs a hint or two on the subject, before he is made aware, that these persons believing themselves to be perfectly sane, cannot help regarding the deprivation of their liberty as an act of high-handed injustice, and the allegation of insanity as a bitter insult added to the injury. Twice only, once in England, and once in France, was I importuned for my assistance in 'getting them out.' I was allowed to pass along, seldom addressed, and exciting a look only of the faintest curiosity. The causes of such a singular phenomenon, I was naturally led to investigate, and, although I may have overlooked some of them, yet I believe, that the principal ones met my attention."

We wish we were able to extract Dr. Ray's exposition of these causes, which is a very interesting one, but our limits forbid us to do more than enumerate them. 1. The proportion of old, incurable, and therefore quieter, cases is far greater in the British and French Asylums than in the American. 2. In these countries, too, insanity seems to far less frequently assume the form of intense and uncontrollable excitement. Common as such cases are in America, Dr. R. did not meet with one in Europe. " The continuance of furious, maniacal excitement for months together, which is so common a circumstance with us, is seldom witnessed in England or France; and nothing which I communicated concerning the disease in this country, excited so much surprise and interest as this trait." Dr. Hutchinson of Glasgow, expressed his belief that cases of intense excitement were much more frequent some years since than at present. 3. The physical condition of the patients in the British Asylums is much less vigorous than is that of the Americans. Want of food is not infrequently described among the causes of the disease in the Reports. 4. The peculiar moral and social condition of the mass of British patients, they being in a state of pauperism, in which they are but too contented with the, to them, luxurious arrangements of an asylum. The American, on the contrary, has been cut short in the course of some hopeful career. 5. The manners existing in European society, and which inculcate so deferential a submission upon the part of the humbler orders, continue to exert their effect upon them with almost the force of an instinct, even when under the influence of their calamities. 6. There is a much smaller proportion of cases of moral insanity, (in which there is a complete perversion of some of the moral sentiments and affections), in the European than in the American hospitals, where they exist in the proportion of 6 in 50, and become *foci* of discontent and confusion among those in whose company they may be chanced to be placed.

11. *Restraint and non-restraint.*—The subject of the disuse of restraint naturally excited much of the author's attention; but the information he was enabled to acquire concerning it was of a very conflicting and unsatisfactory character; and, as with regard to the padded rooms, he arrives at the conclusion that the non-restraint system has been regarded with undeserved favour in Britain, and that insanity is a very different thing here and in America. The directors of the various establishments furnished very discrepant opinions as to the possibility of entirely doing without restraint, especially in the violent description of cases seen in America, and for which none of the substitutes which he has here met with would prove effectnal. We entirely agree with him that cases every now and then occur even in this country for which restraint, not too long continued, affords the most humane as well as the most effectual mode of management. Dr. Ray is disposed to think a disproportionate share of attention has been given to this question.

"When we consider, on the one hand, how slight a thing restraint is in the European institutions, and how seldom it is applied at all; and, on the other, how many points there are in the management of the insane, involving their comfort and curability in a far greater degree, I cannot help concluding that this question has received a degree of attention altogether disproportionate to its intrinsic merits, I do not mean to sanction the idea that the imposition of restraint is an unimportant matter. On the contrary, I would have it regarded as, in most cases, a necessary evil, used only for the prevention of a greater. When insane manifestations are to be resisted, as they sometimes must, the precise form in which it is to be done, whether by mechanical restraint, by seclusion in a room, or by the hands, arms, or legs of attendants, is a question that must be determined by the circumstances of the individual case, not by theoretical considerations."

12. *Labour.*—This the author found actively pursued, and with unanimous testimony in its favour, in most of the European asylums he visited. The very different social position of the patients in America prior to the attack of insanity, already adverted to, prevents this engine of improvement being there so much resorted to. Their independent spirit quite rejects all idea of labouring gratuitously in an institution in which they are detained against their will and at their cost.

13. *Amusements and Recreations.*—The same amusements are observed in the European as in the American asylums. Reading, however, by reason of the frequent illiterate character of the patients, is much less common; and even among the better classes of patients, books, and especially newspapers, are much seldomer seen. Walking and driving out into the country, so frequently practised in America, is seldom permitted in England, where paupers, as a general rule, never go outside the walls.

"There seems to be in the English community an undue apprehension from the insane when at large, The safety of the lieges is thought to require their constant detention within the asylum walls, and the utmost jealousy is manifested of any attempt to relax the rigour of their confinement. One of the Superintendents informed me that on one occasion he permitted a few of his tranquil or convalescent patients, accompanied by attendants, to witness the exhibition of a circus or some dramatic entertainment. The next day to his surprise he was severely censured in the newspapers of the place, for unwarrantably trifling with the safety of the community, by letting loose a band of lunatics. The gratification experienced by these unfortunate fellow-men, and the safeguard provided against any harm were not thought worth taking into account."

The author also severely and justly comments upon the state of public opinion among the wealthier classes in England, which, regarding the occurrence of insanity among any of their members with morbid sensitiveness, as if it were a reflection upon themselves, induces the immuring of these unfortunate beings to a degree not only unrequired by their symptoms, but prejudicial to their recovery. He also observes upon the care with which such patients were removed from his observation when he visited asylums, however deliberately he might be allowed to examine into the condition of the pauper class. While protesting against any desire to exhibit these or any other unfortunate beings to the gaze of heartless curiosity, he is certain the almost monastic seclusion practised is no less injurious. Many of the Superintendents deplored this state of things, but stated that any attempt at its amendment would be followed by the removal of the patients from the establishment.

"It cannot be denied that in every asylum there are some patients who could be made happier and better by occasionally seeing and conversing with their more rational fellow men. The only consideration that should influence our conduct in this matter should be the welfare of the patient, not the selfish feeling of his friends. Intercourse with the world should be regarded in the light of a moral remedy of no insignificant power, which it is as much our right and duty to use where it is evidently beneficial, as to withhold when there is reason to believe its effects would be pernicious. Its management should be left to the Superintendent, who is most able to form a correct opinion on the subject, and his decision ought not to be affected by the squeamishness of friends who would convert an asylum into a prison, nor by the vulgar curiosity of the public who would turn it into a show-house for the exhibition of the saddest infirmities of our nature."

13. *Schools.*—Dr. Ray did not see any schools in operation in the British Asylums, although some were said to exist. In France and America they seem to be in advance of us in this particular, especially in the former country. At the Surrey and Wakefield Asylums much pains have been taken in the instruction of *idiots*. Dr. Corsellis, of the latter institution, has found that, although by perseverance many of their mischievous propensities may be cured, and some simple employment taught them, any attempt to excite intellectual exertion brings on paroxysms of excitement or convulsions. This was not observed at the Bicêtre, where instruction of these unfortunate creatures is pursued on a large scale; and Dr. Ray attributes it to the fact of only the dangerous and diseased idiots being sent to the English asylums, while those of all kinds are sent to the Bicêtre.

14. *Medication.*—As the wonderful effect of enlightened moral treatment became exhibited in the English asylums, the active medical means, heretofore so much in vogue, lost their reputation. A great proportion of the cases in England too are chronic, and very often occur in persons whose supply of food has been defective and innutritious. In such, a supporting treatment is curative which would prove destructive in America; and a far more abundant use of malt liquors is allowable in the one country than in the other. In England, these are often found to subdue excitement, impart a healthy tone to the system, and prepare the way for convalescence beyond any other of the medicinal or dietetic substances more commonly used in America. Narcotics are given in Europe to some extent in order to procure sleep, but not, as in America, for the specific purpose of subduing mental and nervous excitement. For this purpose, the prolonged use of the warm bath or the cold douche is much relied upon in France; and, at Rouen, M. Parchappe employs the warm bath, with cold sponge lying on the forehead, in about sixty patients per diem. On account of its entire proscription in America, the author was much surprised to observe the frequency with which the use of *tobacco* is allowed to the patients in Europe; but he is disposed to believe it, under restrictions, a very proper indulgence.

15. *Religious Exercises.*—This is one of the most delicate of all the points in the moral management of the insane, and one in which well-intending officiousness may do an immensity of mischief.

"That part of the British public which takes any interest in asylums for the insane, is disposed to attach an undue importance to religious exercises in the moral treatment of the insane. They make the common mistake of supposing that, in mental as well as bodily disorder, the patient is equally able and willing to profit by the consolations of religion, while the truth is, that, generally speaking, the insane manifest far less docility than either those who are bound down with bodily infirmity, or those who are sound both in mind and body. *But the insane are sick, and the sick stand in need of religious advice and consolation,* and this logic has led, among other results, to the appointment of *resident chaplains.*

The practice of having a resident chaplain, however, was condemned by the medical officers, without any exception at all. It was thought that to render any essential service in their clerical character—to avoid doing harm indeed—there was required more practical knowledge of insanity, more knowledge of mankind in their sound as well as unsound condition, than the education and habits of clergymen enable them to obtain. * * * * * *

There is another objection to resident chaplains which ought to be conclusive, even were the others much lighter than they really are. It is so difficult to define their exact province in the work of restoring the disordered mind, that collision with the medical officers is not unlikely, nor has it in England been an unfrequent result. The latter may see all their efforts frustrated by what they deem the injudicious course pursued by the chaplain, who, however, cannot be made to regard it in that light: and the consequence is that much ill-feeling is engendered, and a system of unpleasant relations established, productive of incalculable evil. When it so happens that the chaplain is a man of yielding nature, ready always to surrender his own views when conflicting with those of the medical officer, and always willing to make himself useful in any way that can be suggested, then he proves to him a valuable adjunct. But why risk the peace and harmony of an institution upon so uncertain a contingency as the chance of obtaining a person of this description—especially for an object of doubtful utility? It may be said that, if the Superintendent believes the course of the Chaplain to be injudicious, the latter can be replaced by a more suitable person. This is more easily said than done. Every one must be aware that the removal of an officer, however unsuitable, is, at best, a difficult and an unpleasant affair, and consequently, that much is usually suffered before it is undertaken. But leaving all speculative considerations, the success of the arrangement in Great Britain, where it has given rise to much ill-feeling and some scandalous scenes, has not been such as to recommend it very strongly to our favour."

The paper contains some interesting observations upon the disposal of *Criminal Lunatics*, and upon the *Size of Asylums* (Dr. Ray not approving of the very large ones in America), but our want of space prevents our adverting to them. As it is, we have noticed the Essay at greater length than we intended; but it is written in so fair and candid a spirit, that we thought it desirable to present our readers with a pretty full account of its contents as a *pendant* to the article on Insanity in our last Number.

